# Fleeing lockdown and its impact on the size of epidemic outbreaks in the source and target regions – a COVID-19 lesson

**DOI:** 10.1038/s41598-021-88204-9

**Published:** 2021-04-29

**Authors:** Maria Vittoria Barbarossa, Norbert Bogya, Attila Dénes, Gergely Röst, Hridya Vinod Varma, Zsolt Vizi

**Affiliations:** 1 Frankfurt Institute for Advanced Studies, 60438 Frankfurt, Germany; 2 Bolyai Institute, University of Szeged, Szeged, 6720 Hungary; 3 Interdisciplinary Center for Scientific Computing, 69120 Heidelberg, Germany

**Keywords:** Viral infection, Applied mathematics

## Abstract

The COVID-19 pandemic forced authorities worldwide to implement moderate to severe restrictions in order to slow down or suppress the spread of the disease. It has been observed in several countries that a significant number of people fled a city or a region just before strict lockdown measures were implemented. This behavior carries the risk of seeding a large number of infections all at once in regions with otherwise small number of cases. In this work, we investigate the effect of fleeing on the size of an epidemic outbreak in the region under lockdown, and also in the region of destination. We propose a mathematical model that is suitable to describe the spread of an infectious disease over multiple geographic regions. Our approach is flexible to characterize the transmission of different viruses. As an example, we consider the COVID-19 outbreak in Italy. Projection of different scenarios shows that (i) timely and stricter intervention could have significantly lowered the number of cumulative cases in Italy, and (ii) fleeing at the time of lockdown possibly played a minor role in the spread of the disease in the country.

## Introduction

A novel coronavirus (SARS-CoV-2) causing the severe acute respiratory illness COVID-19 appeared in China at the end of 2019. The first cases were identified in Wuhan, a city with over 11 million inhabitants, capital and largest city of Hubei Province. Wuhan being the political, economic, financial, commercial, cultural and educational centre of Central China and a major domestic and international transportation hub, it was expected that the disease could easily spread to other parts of China and to other countries. On January 23, 2020, in an attempt to control the spread of the disease, the Chinese government introduced a lockdown in Wuhan and other cities in Hubei. The residents of Wuhan were informed at 2 am that from 10 am of the same day, all public transport would be suspended. Wuhan residents would thereafter not be allowed to leave the city without permission. This notice was followed by an exodus from Wuhan. It was reported that around 300,000 people left Wuhan by train alone before the start of the lockdown^1^. It is important to note that prior to the lockdown of Hubei on January 23, almost all detected cases in other major cities were exported from Wuhan^2^.

Similar events happened in Italy, where the first lockdown measures were introduced on February 21, with ten municipalities of the province of Lodi (Lombardy) and one in the province of Padua (Veneto) being quarantined^3^. This measure affected around 50,000 people. Extension of the quarantine zone to a large area of Northern Italy, including the whole region of Lombardy and 14 major provinces in Emilia-Romagna, Veneto, Piedmont and Marche, was announced on March 8. Travel from and to the affected areas was restricted, though not completely banned. A leakage of a draft of the decree on the night of March 7, published by *Corriere della Sera*, resulted in a panic in the affected areas. Major newspapers reported of hundreds of people crowding onto trains and buses to leave the extended quarantine area in Milan^4^. Overall, thousands of people reportedly left the north for the southern regions, including Sicily^5^ and Sardinia^6^.

A few mathematical studies on the Chinese data have investigated the effects of mobility from quarantined regions from different points of view. Chen et al.^7^ analysed the correlation of case numbers and population migration data of Wuhan and Hubei to find that the number of infected cases was highly correlated with the emigrated populations from Wuhan. Li et al.^8^ applied a nonautonomous mechanistic model to study the effects of the lockdown and predict the virus transmission in Wuhan and Beijing. Most studies (see, e.g. Zhao et al.^2^, Lau et al.^9^, Lai et al.^10^, Boldog et al.^11^) investigate the association between the domestic/international travel load and the number of cases exported from Wuhan to other regions in China. These studies, however, do not seem to consider the large exodus induced by the lockdown measures.

We present here a mathematical framework for studying the effects of a potential lockdown-induced exodus, together with the time and strength of intervention measures, on the outbreak size in different geographic regions. We assume that the outbreak starts in one specific area, simply denoted as *region A*, where lockdown is implemented at time *T*. This induces people from region A to flee the lockdown and move to another geographic *region B*. If region B was disease-free up to time *T*, the lockdown-induced migration from region A can lead to importation of the infection in a previously uninfected area. We formulate and study a compartmental epidemic model for the spread of an infectious disease in the two different geographic regions, establishing a new type of intervention-dependent final size relation. The latter can be used to estimate the final epidemic size even in the case of a change in the population size due to the migration just before the lockdown. This model is calibrated on the example of the Italian outbreak. By means of numerical simulations we project different scenarios showing that (i) timely and stricter intervention could have significantly lowered the number of cumulative cases in Italy, and (ii) migration of at the time of lockdown possibly played a minor role in the spread of the disease in the country. The approach presented in this study can be adapted to describe the spread of an infectious disease over multiple geographic regions or to describe the transmission of viruses others than SARS-CoV-2.

## Methods

### Modeling transmission dynamics

The mathematical model proposed in this study is based on a system of ordinary differential equations (ODEs) that describes interactions between different groups of individuals in the population. The proposed approach extends the known *S-E-I-R* (susceptibles–exposed–infected–recovered) model for disease dynamics^12^, and is suitable to describe the spread of several infectious diseases, though we shall specifically talk about COVID-19 later. Before describing the lockdown scenario and other intervention measures aimed at mitigating the outbreak, we introduce the core model for the (uncontrolled) spread of the virus in a population. The ODE approach that we use assumes that the population is homogeneous and well-mixed within a region. Individuals are classified according to their status with respect to the virus spread in the community.

**Figure 1:**
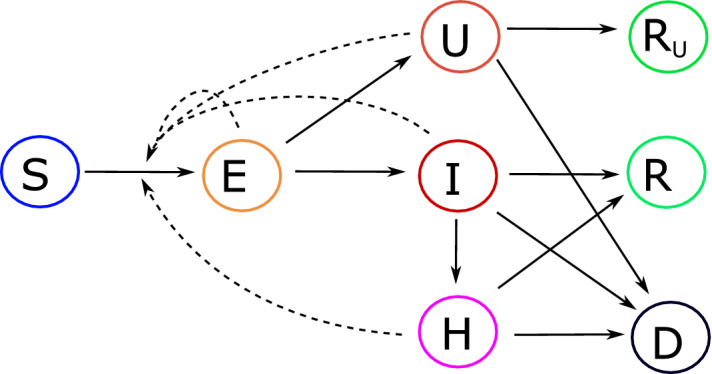
Model structure for the transmission dynamics of an infectious disease, on the example of COVID-19. Solid arrows indicate transition from one compartment to another, dashed arrows indicate virus transmission due to contact with infectives. Upon infection, susceptible (*S*) individuals enter a latent/presymptomatic phase (*E*). After symptoms onset, infections may be detected (*I*) or remain undetected (*U*). Detected cases might become severe and require hospitalization (*H*). Infected individuals who recovered from a detected (*R*) or an undetected ($R_U$) infection, as well as patients who died (*D*) upon infections, are removed from the chain of transmission.

At the beginning of the epidemic, that is, when first cases are reported, the majority of the population is assumed to be susceptible (*S*), and hence can be infected. The time between exposure to the virus (becoming infected) and symptom onset, on average $1/\alpha$ days, is known as “exposed” or “presymptomatic” period. To better understand the role of transmission from infected people with mild or no symptoms, we distinguish between transmission from people who are infected, might have very mild symptoms, but remain undetected (*U*) and transmission from people who are infected but still in the presymptomatic period (*E*). Detected infectives might require hospitalization (*H*) or not (*I*). Detection of cases is assumed to occur either after the latent phase (with probability $\rho$) or post mortem (with probability $\sigma$). Duration of infection can be different for the three different groups. Undetected infections lead to undetected recoveries, which cannot be reported unless testing for ongoing (virus detection) or previous (antibody detection) infections is performed. Individuals who recovered from a detected (*R*) or an undetected ($R_U$) infection, as well as patients who died from the infection (*D*), are removed from the chain of transmission. Separation of the compartments *R* and $R_U$ allows to keep track of how many people have recovered from infection without having been detected. At the same time, it is possible to use *R*(*t*) for model calibration, when time series for recovered infectives are sufficiently reliable. Susceptible individuals can be infected via contacts with presymptomatic (transmission rate $\beta _E$), undetected cases (transmission rate $\beta _U$), detected but not hospitalized ($\beta _I$) and hospitalized cases ($\beta _H$). We assume that undetected infectives, due to possibly absent or unspecific symptoms, do not restrict their contacts to others, and therefore have higher transmission rates than detected infected individuals. Hospitalized cases are properly isolated, and hence their transmission rate is assumed to be the lowest. The interpretation of model parameters, all assumed to be nonnegative, is summarized in Table 1. The dynamics of the core model described above and shown in Fig. 1 is given by the following system of differential equations:

1 $$\begin{aligned} \begin{aligned} 
\dot{S}(t)&= -\lambda (t) S(t)&\text{ susceptibles},\\ 
\dot{E}(t)&= \lambda (t)S(t) - \alpha E(t)&\text{exposed/presymptomatic},\\
\dot{U}(t)&= (1-\rho )\alpha E(t) - \gamma _{U} U(t)&\text{ undetected infectives},\\
\dot{I}(t)&=\rho \alpha E(t)-(\gamma _I +\delta _I +\eta ) I(t)&\text{ detected, non-hospitalized infectives},\\
\dot{H}(t)&= \eta I(t)-(\gamma _H +\delta _H) H(t)&\text{ hospitalized infectives},\\
\dot{R}(t)&=\gamma _I I(t)+\gamma _H H(t)&\text{recovered from detected infection},\\
\dot{R}_U(t)&= (1-\sigma )\gamma _U U(t)&\text{recovered from undetected infection},\\ 
\dot{D}(t)&= \delta _I I(t)+\delta _H H(t)+\sigma \gamma _U U(t)&\text{deceased}, 
\end{aligned} \end{aligned}$$

where

$$\begin{aligned} \lambda (t) = \frac{{\beta _E E(t)+}\beta _I I(t)+\beta _U U(t) +\beta _H H(t)}{{N(t)}}, \end{aligned}$$

and $N(t)= N_0-D(t)$, $N_0$ being the total initial population at the beginning of the outbreak. That is, $N(t)=S(t)+E(t)+U(t)+I(t)+H(t)+R(t)+R_U(t)$ is the current “effective” population at time *t*. Demographic variations other than disease-induced deaths are not considered in this work.

**Table 1:** Interpretation of model parameters.

Parameters	Description (unit)
$\beta _E$	Transmission rate from presymptomatics to susceptibles (1/(days × contact))
$\beta _U$	Transmission rate from undetected infectives to susceptibles (1/(days × contact))
$\beta _I$	Transmission rate from non-hospitalized cases to susceptibles (1/(days × contact))
$\beta _H$	Transmission rate from hospitalized cases to susceptibles (1/(days × contact))
$1/\alpha$	Duration of latency period (days)
$\gamma _U$	Recovery rate for undetected infectives (1/days)
$\gamma _I$	Recovery rate for non-hospitalized cases (1/days)
$\gamma _H$	Recovery rate for hospitalized cases (1/days)
$\rho$	Probability of detection on symptoms onset
$\sigma$	Probability of detection post mortem
$\eta$	Hospitalization rate (1/days)
$\delta _I$	Disease-induced death rate for non-hospitalized cases (1/days)
$\delta _H$	Disease-induced death rate for hospitalized cases (1/days)
$\phi$	Fraction of unconstrained population migrating from region A to region B
*T*	Time of lockdown of region A

### Numerical integration, model calibration and sensitivity analysis

**Figure 2:**
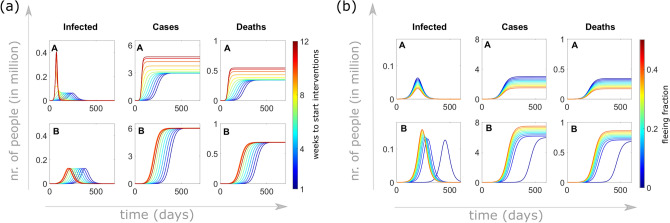
Detected infected cases and deaths depending on (**a**) the lockdown time ${\varvec{T}}$ and (**b**) the fleeing fraction of exposed/undetected individuals at the time of the lockdown. An initial outbreak starts off with 20 cases and two deaths in region A (population 25 million), where lockdown is established at time *T*, immediately followed by lockdown-induced migration of a fraction of the unconstrained population from region A to the disease-free region B (population 50 million). (**a**) The lockdown time *T* varies from one to twelve weeks after the initial reporting, while the fleeing fraction is fixed to 1% of the unconstrained population in region A; (**b**) 0.01% to 50% of the unconstrained population moves from region A to region B at the fixed time $T=21$ days after the initial reporting. At the time of lockdown, control measures are applied in both regions in order to restrict contacts by 60% and reduce transmission.

**Figure 3:**
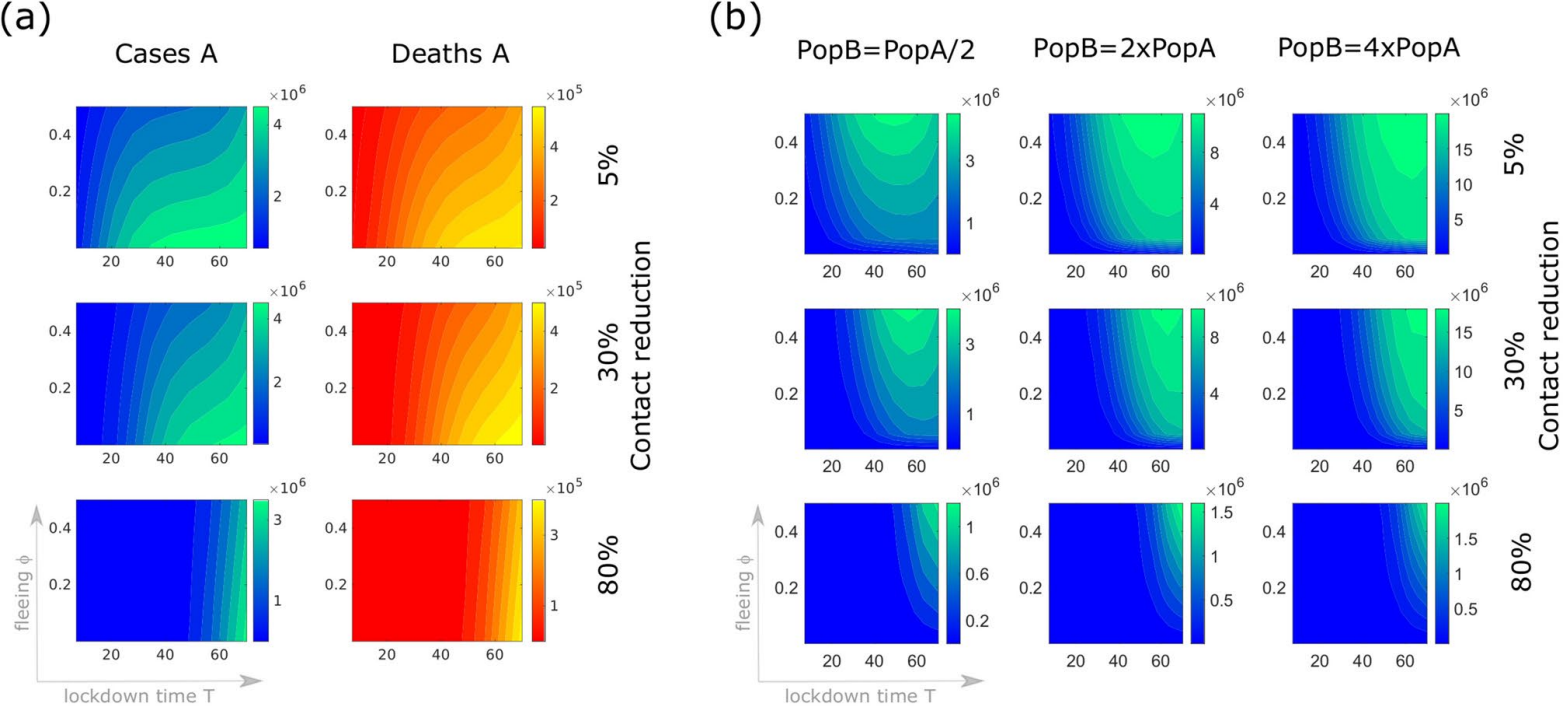
Sensitivity of cases in region B depending on the lockdown time ${\varvec{T}}$, the fraction $\varvec{\phi }$ of population leaving region A, the strength of intervention measures in reducing contacts, and the population size of region B. An initial outbreak starts off with 20 detected cases and two deaths in region A (population 25 million) which is locked at time *T* (horizontal axes indicate days after the initial reporting) and lockdown-induced migration (vertical axes) occurs. At the time of lockdown, control measures are applied to restrict contacts by 5% (first row), 30% (second row) or 80% (third row), and maintained for two months. Color codes represent cases/deaths projected at the end of the two months following model (1): (**a**) the number of detected cases (left column) and deaths (right column) in region A, (**b**) detected case numbers in region B depending on the size of the population in region B with respect to that of region A (columns).

**Figure 4:**
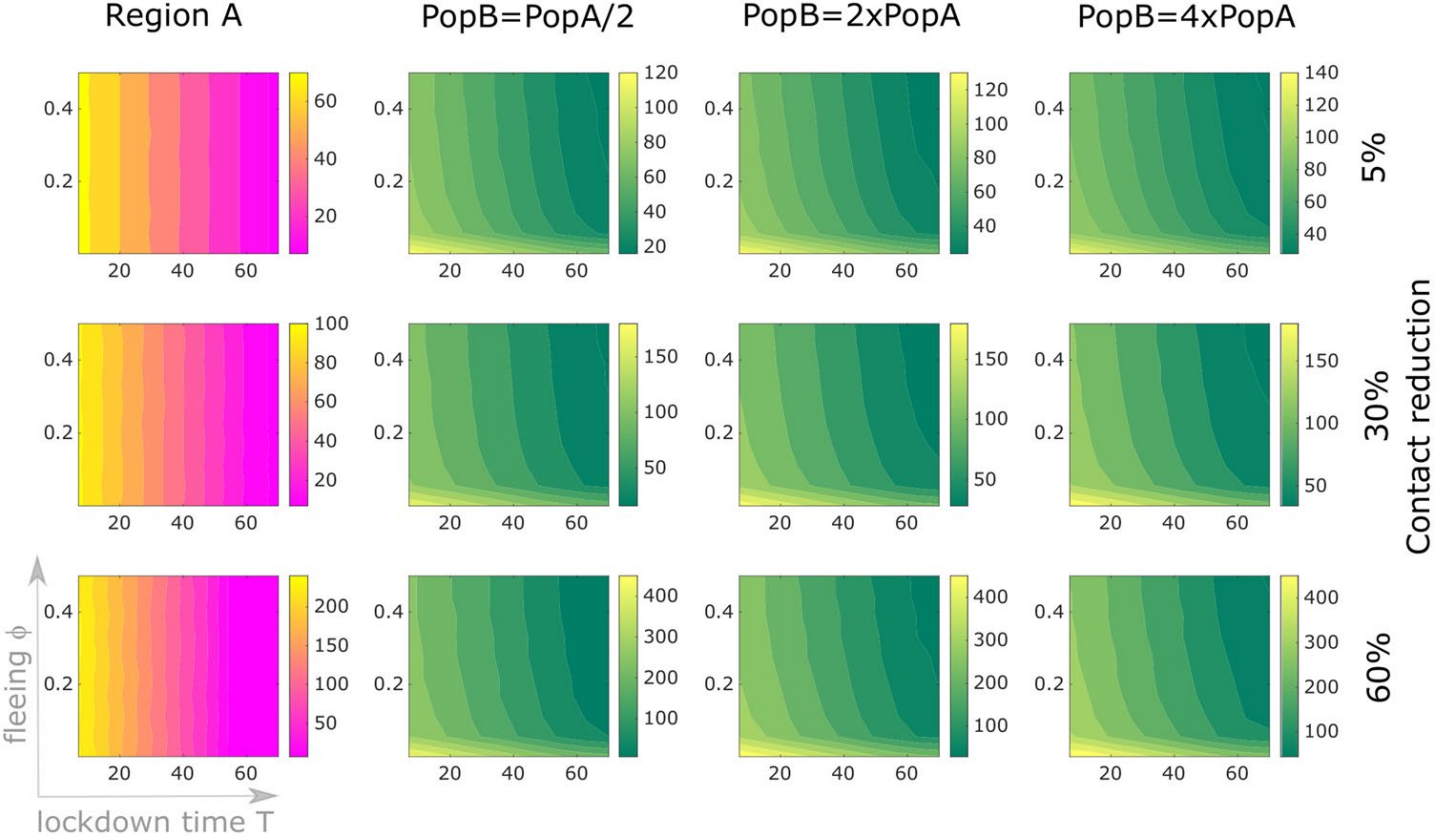
How long should control measures be in place? An initial outbreak starts off with 20 cases and two deaths in region A (population 25 million) which is then locked at time *T* (horizontal axes indicate days after the initial reporting) and lockdown-induced migration (vertical axes) occurs. In both regions, at the time of lockdown, control measures are applied to restrict contacts by 5% (first row), 30% (second row) or 60% (third row), and maintained for 7 days after the peak in the daily incidence in the respective region is reached. Color codes represent the duration in days of the control period in region A (first column) and region B (second to fourth columns), also depending on the population size in region B (being half, twice or four times the population in region A).

**Figure 5:**
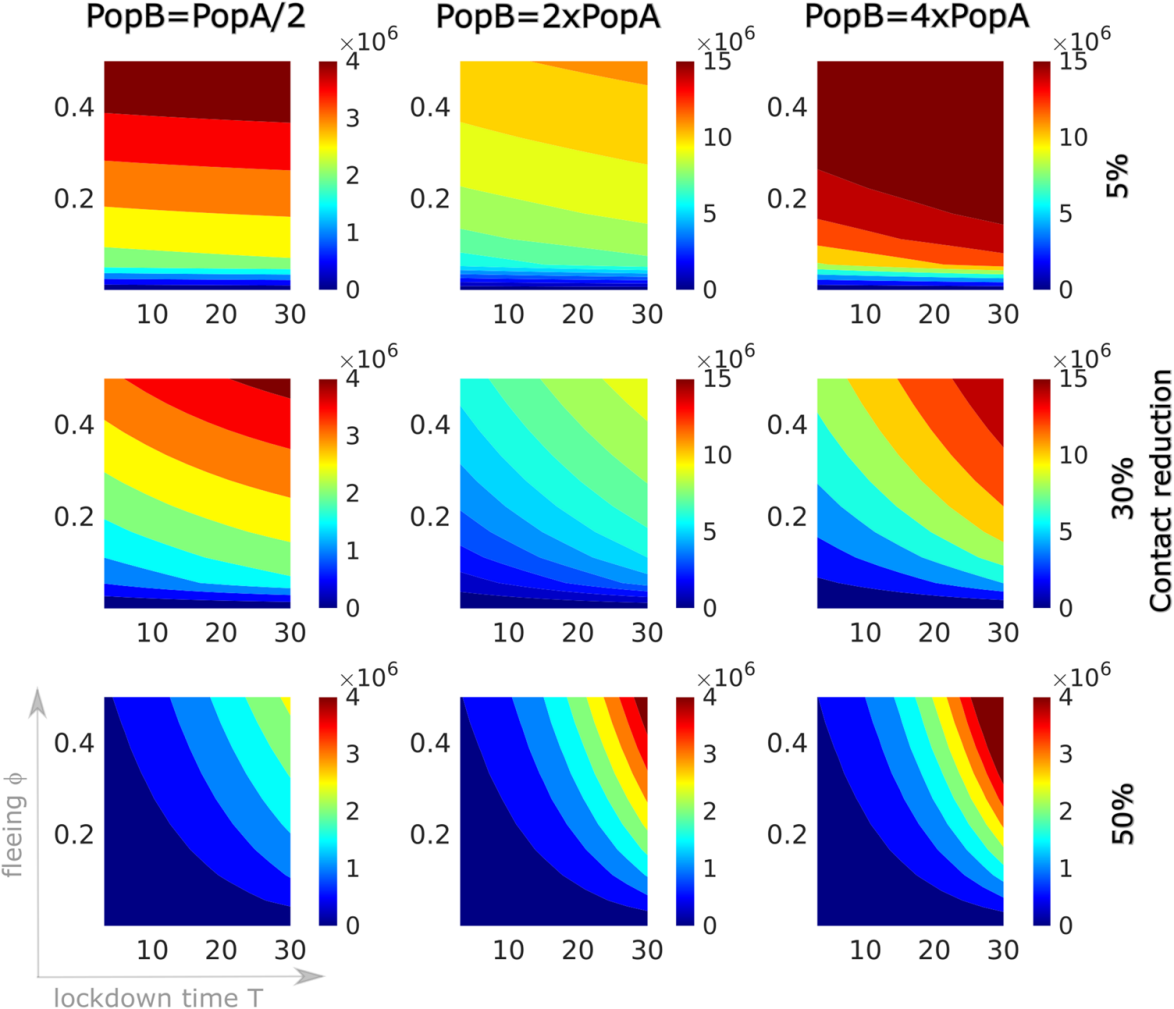
Cumulative detected cases in region B three months after lockdown of region A depending on the fraction $\varvec{\phi }$ of population leaving region A, the intervention time $\varvec{T_B}$ in region B, the effectiveness of intervention measures in reducing contacts, and the population size of region B. Region A is isolated 21 days after the beginning of the outbreak and migration towards region B occurs. For each panel, the vertical axis denotes the variation in the fleeing fraction from A ($\phi$, between 0.1% and 0.5). On the horizontal axis, the reaction time $T_B$ indicates how many days passed for control measures in region B to be applied since isolation of region A. Control measures are applied in region B to restrict contacts by 5% (first row), 30% (second row) or 50% (third row), and maintained until the end of the simulations. Cumulative detected cases are projected also depending on the size of population in region B with respect to that in region A (columns). Note the different scaling of the color legend in the panels.

The functionalities for solving the epidemic model (1) and fitting the parameters to the data were implemented in Python language: we use odeint function from the scipy.integrate package^13^ for integrating the model equations and the non-linear least-squares minimization from the lmfit module^14^ for estimating the parameters. We selected the L-BFGS-B method^15^ for the optimization procedure, which is a very efficient algorithm for solving large scale problems. In case the time series were incomplete, the days corresponding to the missing values were omitted in the minimization. For all methods, we used the default values for options such as tolerances and step sizes. The confidence intervals of the estimated model parameters were computed based on the standard error of the estimated covariance matrix. For the plots in Figs. 2, 3, 4, and 5, integration in MATLAB ^®^ by means of available ODE routines (ode45, ode23s) was used. Numerical approximation of the final size formula was verified by comparing the numerical solution of the implicit equation (4) (computed using fsolve from the scipy.optimize package) with the numerical integration of the ODE model (1) over a sufficiently long time interval (cf. Supplementary Material). Parameter sensitivity analysis of model quantities of interest, such as the basic reproduction number, the number of hospitalized cases at the outbreak peak and the final size, were investigated by means of Partial Rank Correlation Coefficients (PRCC) analysis ^16^. Latin Hypercube Sampling ^17^ was used to generate a representative sample set of tuples of parameters from the parameter ranges indicated in the Supplementary Material. Using PRCC, we can rank the effect that each parameter has on the outcome, when other parameters are simultaneously varying in the given ranges. Calculation of PRCC allows us to determine the strength of statistical relationships that exist between each input parameter and the output variable. Parameters with PRCC larger (respectively, smaller) than zero are positively (respectively, negatively) correlated with the quantity of interest.

## Results

### Reproduction number

Possibly among the most interesting quantities to identify in the early phase of an outbreak, the *basic reproduction number* (denoted by ${\mathcal {R}}_0$) is used in mathematical epidemiology as an indicator for the transmissibility of the disease. This ${\mathcal {R}}_0$ is a metric which indicates the average number of secondary infections generated in a fully susceptible population by one infected host over the course of the infection. In most epidemiological models, ${\mathcal {R}}_0>1$ leads to a disease outbreak, whereas for ${\mathcal {R}}_0<1$ the disease will not spread in the population.

The basic reproduction number of system (1) can be calculated analytically (cf. Supplementary Material), e.g., by means of the next-generation matrix approach^18^, and is given by

2 $$\begin{aligned} {\mathcal {R}}_0=\underbrace{\frac{ \beta _E}{\alpha }}_{=:{\mathcal {R}}_0^E}+\underbrace{\frac{\rho \beta _I}{\gamma _I+\delta _I+\eta }}_{=:{\mathcal {R}}_0^I} + \underbrace{\frac{\rho \beta _H\eta }{(\gamma _I+\delta _I+\eta )(\gamma _H+\delta _H)}}_{=:{\mathcal {R}}_0^H} + \underbrace{\frac{ \beta _U (1-\rho )}{\gamma _U}}_{=:{\mathcal {R}}_0^U}. \end{aligned}$$

The summands of ${\mathcal {R}}_0$ account for contacts with presymptomatic cases (${\mathcal {R}}_0^E$), undetected infectives (${\mathcal {R}}_0^U$), detected non-hospitalized (${\mathcal {R}}_0^I$) and hospitalized (${\mathcal {R}}_0^H$) cases. The reproduction number evolves in time with time-dependent parameters, which might vary e.g. because of intervention measures, and with variations in the susceptible portion of the population. As the outbreak evolves, people who go through infection are either immunized or die, and the susceptible population decreases (vaccination-induced immunity is not considered here but would also contribute to lowering the susceptible population). The *effective reproduction number* ${\mathcal {R}}_t$ at time *t* can be obtained by the same formula, substituting the values of time-dependent parameters in (2) and multiplying this value by the susceptible fraction of the population, *S*(*t*)/*N*(*t*). The reproduction number can be controlled via intervention measures aiming not only at reducing transmission, but also at improving detection, or at enhancing isolation and treatment of infectives. Figure S1 in the Supplementary Material shows that the reproduction number can be most effectively controlled via regulation of transmission rates.

### Modeling the lockdown at time ${\varvec{T}}$ and migration from region A

Inspired by the history of the COVID-19 outbreak in China and Italy, we use the previously presented transmission network (1) to describe an outbreak spreading from one geographic region A, to the rest of the country (here considered as one single region, B). The country itself is assumed to be isolated, that is, migration inward/outward is not considered here. The outbreak is assumed to have started in region A, that is, numerous cases were detected in a short time during the early phase of the epidemic. The prevalence (cases/population) of COVID-19 in region A being much higher than in the rest of the country (region B), the government decides that region A is going to be confined at some time *T*. This motivates many people to move from region A to region B, just before region A is effectively isolated from the rest of the country. We adapt the mathematical model (1) to reproduce this situation and understand how the lockdown time *T*, and the migration of a fraction $\phi \in (0,1)$ of the *unconstrained population* (that is, everyone except detected, hospitalized and deceased persons) from region A to region B could affect the evolution of the epidemic in the two regions. Detected cases, hospitalized cases and deceased individuals, cannot leave region A at any time. Transitions between region A and region B, which are assumed to be in balance before the lockdown, are stopped after time *T*. Migration is modeled as an impulse at time *T*, simplifying a possible continuous transition from A to B in the days immediately ahead the lockdown. This means we impose the conditions

3 $$\begin{aligned} {} & \begin{cases}Z^B(T_+)&=\phi Z^A(T_-)+Z^B(T_-)\\Z^A(T_+)&=(1-\phi ) Z^A(T_-)\end{cases}         \quad  \text{ for }\, Z=S,E,U,R,R_U, \; \text{ and }\\[0.1cm] & \phantom{ mm } X^{j} (T_+) = X^{j} (T_-),\qquad \qquad \text{ where } \, X = I,H,D, \;\; j=A,B,\end{aligned}$$

where $T_-$ denotes the time just before migration occurred and and $T_+$ denotes the time of migration, respectively.

After the lockdown ($t\ge T$) the outbreak evolves independently in the two regions. Further, concurrent with the lockdown, other non-pharmaceutical intervention measures, such as encouraged social distancing, school closure, shut down of many economic activities, increased testing activity, improved hospital capacity etc., might be introduced in both regions, possibly in a different manner or with different timing.

### Final size

The final size relation is an analytic formula to predict the total number of individuals in the population who are infected over the course of the epidemic^12^. By means of analytical methods (cf. Supplementary Material), we derived a final size relation taking into account the lockdown time (*T*) and the induced exodus at the time of the lockdown. This formula provides a reliable approximation of the cumulative cases, when the total population and the parameters do not vary too much after time *T*. Assume the status of the system, including the numbers of cases, hospitalizations, deaths, recoveries, and active population (${{\mathcal {N}}}$), in one region right after the lockdown time (denoted below by the subscript ${T{_+}}$) can be obtained from available data or approximated by means of the model (1). Then the final size formula

4 $$\begin{aligned}\ln S_{T{_+}}- \ln S_{\infty }={}& \Phi ({{\mathcal {N}}}-S_\infty ) + {\tilde{b}}_1(\chi - 1 ) I_{T{_+}} + (\chi -1){\tilde{b}}_2 H_{T{_+}}\\& + \left( \chi ({\tilde{b}}_3 -\tilde{a}_4) + \Phi -{\tilde{b}}_3\right) D_{T{_+}} \\&+ (\chi ({\tilde{b}}_4 - \tilde{a}_2)-{\tilde{b}}_4) R_{T{_+}} - \chi \tilde{a}_1{U}_{T{_+}}-\chi \tilde{a}_3{R}^U_{T{_+}}, \end{aligned}$$

allows us to determine the number of cases occurring over the complete course of the outbreak, without requiring model integration after time *T*. Observe that the fleeing ($\phi$) is included in the relation  (4) into ${{\mathcal {N}}}$ and the terms with ${T{_+}}-$subscript, following the conditions (3). Here $S_{\infty }$ denotes the portion of population which remains susceptible at the end of the outbreak. If $S_0\approx N_0$ is the susceptible (total) population at the beginning of the outbreak, then $S_0-S_{\infty }$ corresponds to the cumulative number of cases at the end of the outbreak. The coefficients $\chi ,\, \Phi ,\,{\tilde{a}}_j,\, {\tilde{b}}_j$, $j=1,\ldots , 4$ in equation (4) are obtained from the parameters of model (1) as detailed in the Supplementary Material. Formula (4) is used below to analyze the sensitivity of the total number of cases with respect to model parameters.

### How lockdown and intervention measures affect the evolution of the outbreak

The proposed model (1) with the lockdown scenario (cf. “Methods”) is used to investigate the sensitivity of case and death counts with respect to the intervention parameters, such as the time of lockdown of the source region A, the fraction of population fleeing region A due to the lockdown announcement, and the strength and timing of intervention measures in both regions. We consider a hypothetical scenario which is suitable to describe various infectious diseases and populations, though inspired by COVID-19 spreading in a fully susceptible population. Region A, where the outbreak starts, is assumed to have a population of 25 million. Though the population size in region B can vary, this region is always assumed to be disease-free until lockdown of region A occurs. Region B is parametrized as region A, unless explicitly mentioned. We set the parameter values such that the initial reproduction number in region A is ${\mathcal {R}}_0 \approx 3.8$, while the *beginning of the outbreak* is defined to occur with 20 cumulative detected cases and two deaths. For further details on the parametrization used for this section, we refer to the Supplementary Material.

Figure 2a shows how the cumulative number of detected cases and deaths depend on the lockdown time *T*. At the time of lockdown, which varies here between one and twelve weeks after the beginning of the outbreak in region A, 1% of the unconstrained population flees from region A to region B. Lockdown is coupled with stronger control measures, both in region A and region B, which assume reduction in contact rates by 60% to be maintained until the end of the observations (here about two years after beginning of the outbreak). This reduces the reproduction number in both regions, but not enough to prevent an outbreak also in region B (${\mathcal {R}}^B_T\approx 1.5$). The panels in Fig. 2a show that in both regions, the time of the lockdown strongly affects both the number of cases and the peak of the epidemic wave. Figure 2b shows the cumulative number of detected cases and deaths, depending on the fraction of exposed and undetected infected individuals leaving region A for region B. Here the lockdown time *T* is fixed at three weeks after the beginning of the outbreak, whereas the migrating population $\phi$ varies (between 0.01% and 50% of the unconstrained population in region A at the lockdown time). Increasing the fleeing fraction $\phi$, the peak in cases decreases in region A, whereas in region B, it increases and advances in time.

Figure 3a shows the variation in cumulative detected cases and deaths in region A depending on the lockdown time *T*, the fraction $\phi$ of population leaving region A and the effectiveness of intervention measures in reducing contacts. We project here the number of cases and deaths predicted by the model two months after lockdown, while control measures are maintained for the whole period. While migration does not particularly affect the evolution of the outbreak in region A in case of early intervention, the later the lockdown and the weaker the control measures, the higher will be the number of cases and deaths. In case of late lockdown time (about two months after the beginning of the outbreak), a significant fleeing population reduces the number of cases/deaths in region A, in agreement with what observed in Fig. 2. Opposite is the effect of migration from region A on cumulative cases (and deaths, not shown here) in region B. Fig. 3b shows the importance of applying stronger control measures in region B when region A is locked. When contact restrictions are more severe, the size of the population of region B does not importantly affect the outcome of the outbreak. If contact restriction is minimal in region B, a higher populated target region allows a longer and more dramatic outbreak (with more cases and more deaths).

One of the most difficult questions to answer during an ongoing epidemic concerns the duration of restrictive control measures. How long should these restrictions be in place? Several criteria could be applied to determine the end of the restrictive measures^19^. Here we assume that a region is released one week after reaching the peak in the daily incidence of reported cases. As in the above simulations, we assume that the outbreak spreads from region A to region B when the former is isolated. The result is shown in Fig. 4. In both regions, at the time of lockdown, control measures are applied to restrict contacts by 5% to 60%, and maintained for 7 days after the peak in the daily incidence in the respective region is reached. More restrictive measures postpone (and lower, cf. Fig. 3b) the peak of the outbreak, however this prolongs the time that the control measures should be in place. On the other hand, migration and late intervention accelerate the speed of the epidemic in region B, and hence the peak comes earlier (is much higher, brings more cases and more deaths, cf. Fig. 2) and makes the lockdown time shorter.

So far we have assumed that control measures in region B are applied when region A is isolated. Figure 5 illustrates the damage that could be induced by a delayed reaction in region B combined with the application of not sufficiently strong control measures. The panels show the number of cases in region B three months after lockdown of region A occurred (here *T* is fixed on day 21 since the beginning of the outbreak), assuming that the interventions in region B is delayed by $T_B$ days with respect to region A.

## COVID-19 in Italy as an example

In the previous section we used the model (1) to study the spread of a potential outbreak on two hypothetical geographic regions, without specific application to real data. Here we consider the recent COVID-19 outbreak in Italy and apply model (1) to study the lockdown and the early outbreak dynamics. The COVID-19 outbreak hit Italy in early 2020 as the first country in Europe. While preliminary control measures were introduced locally at the end of February as first cases were detected, at the beginning of March a large area including Lombardy and several provinces in Emilia-Romagna, Veneto, Piedmont and Marche were declared as “red zone” and isolated^20^. Shortly after, even more restrictive control measures were applied on the whole country^3^.

### Data and setting for parametrization

The publicly available data-set provided by the Italian Ministry of Health (Ministero della Salute) and the national Civil Protection Department (Dipartimento della Protezione Civile)^21^ was used for this study. Time series for hospitalized (totale_ospedalizzati), daily case incidence (nuovi_positivi) and cumulative cases (totale_casi), as well as deaths (deceduti) were obtained from the *dati-regioni* repository^21^. Data for region A was prepared accounting for the isolated provinces^3,22^. As hospitalization and death counts are available at regional level, but not at the level of provinces, we define region A as the area composed of the whole regions of Lombardy, Emilia-Romagna, Veneto, Piedmont and Marche (about 25 million people). In reality, only fourteen largely-neighboring provinces in Emilia-Romagna, Veneto, Piedmont and Marche were isolated, together with Lombardy which is the most populous region in Italy and accounts for the majority of COVID-19 cases. Region B is defined as the rest of the country (about 35 million people). The model is calibrated on daily incidence, deaths and hospitalized time series for the two regions. Based on estimates from previous studies, we fix the average duration of the latent phase ($1/\alpha \approx 5.5$ days^23,24^), and the duration of the infectious periods in undetected (ca. 7 days^24,25^) and in detected cases (ca. 10 days^26,27^). For model parameters that cannot be obtained from other references nor estimated reliably with the available data, we fix values based on plausible assumptions. For example, we fix the ratio between $\beta _E$ (respectively, $\beta _I$ and $\beta _H$) and $\beta _U$ and estimate the latter. It is unclear which amount of secondary cases resulted from presymptomatic transmission, with estimates ranging from 6.4%^28^ to 44%^29^. Here we assume that $\beta _E=0.2\beta _U$, accounting for a two days infectious period in the presymptomatic phase. As undetected infectives have possibly mild or no symptoms, we assume that they do not restrict their contacts to others, and therefore have higher transmission rates than detected cases ($\beta _I=0.25 \beta _U$). Hospitalized patients are supposed to have very limited contacts compared to undetected cases ($\beta _H=0.1 \beta _U$). The detection ratio $\rho$ is estimated based on Table 1 of the very recent report by the Italian National Institute of Statistics and the Ministry of Health on the seroprevalence in Italy^30^. From the data shown in this report, we could compute a seroprevalence 4.53% (weighted average) for region A and 0.96% for region B, as of July 20, 2020. As of the same date, the cumulative cases in region A and region B report about 182,000 cases detected in region A and about 62,000 cases detected in region B^21^. Rounding the seroprevalence to 5% for region A, this leads to 1.25 million estimated COVID-19 cases for region A, out of which we know only 182,000, henceforth a detection ratio $\rho _A\approx 14.5\%$. Analogously, rounding to 1% seroprevalence for region B, we obtain $\rho _B\approx 17.5\%$. We assume that the detected cases are hospitalized with a rate $\eta =p_H/2$, where the probability $p_H\in (0,1)$ of being hospitalized two days after symptoms onset is estimated from the data. Once the detected cases are hospitalized, they are assumed to either recover with a probability $p_{HR} \in (0, 1)$ after 10 days, or die with a probability $(1 - p_{HR})$. This leads to a recovery rate of $\gamma _H=p_H p_{HR}/10$ in the hospitals and a death rate of $\delta _H=p_H (1-p_{HR})/10$. The probability $p_{HR}$ is estimated from the data. Among those detected cases that do not get hospitalized, we assume that $p_{IR}=99\%$ of the cases recover after on average 10 days from symptoms onset, while the remaining 1% die. This means that we can interpret the recovery rate of the detected cases that are not hospitalized as $\gamma _I=(1 - p_H) p_{IR}/10$ and the death rate as $\delta _I=(1 - p_H)(1- p_{IR})/10$. We assume that $\sigma =0.1\%$ cases are detected post mortem.

The initial time point for simulations is set to February 24, 2020. Most of the initial conditions for solving the ODE system, such as the number of deaths or hospitalized cases could be obtained directly from the data. However, there is no data available for the exposed and undetected infections. These values are approximated making use of the detection ratio $\rho$ and the average latency period $1/\alpha$, with $\rho E(0)$ roughly corresponding to the case incidence six days later, and *U*(0) corresponding to the undetected fraction $(1/\rho -1)I(0)$ of cases at the beginning of the observations. The initial conditions used for simulations are specified in the Supplementary Material.

**Figure 6:**
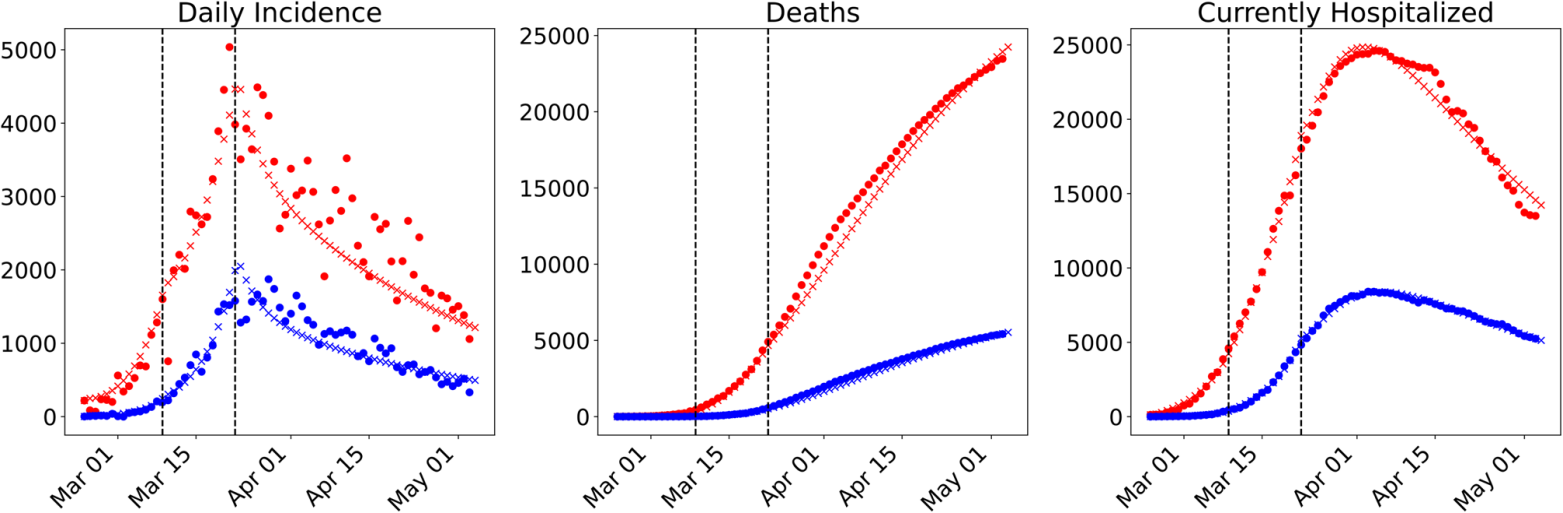
Model fit for the early dynamics of the COVID-19 outbreak in Italy. Reported data (dots) and model results (crosses) for daily incidence (left panel), deaths (middle panel) and hospitalized cases (right panel) in region A (Lombardy, Emilia-Romagna, Marche, Piedmont, Veneto; red) and region B (rest of the country; blue). Parameter values are estimated as indicated in three different time intervals (separated by vertical lines in the figure): pre-lockdown (February 24–March 8), first lockdown measures (March 9–21), and extended lockdown measures (March 22–May 4), cf. Supplementary Material.

**Figure 7:**
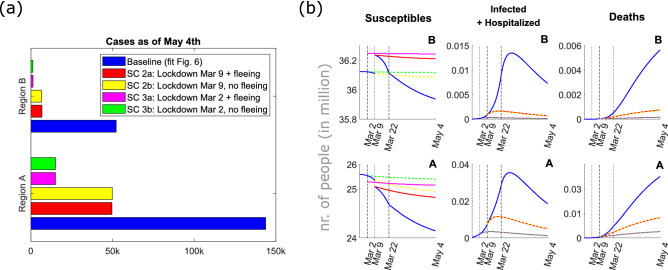
Scenario comparison for (**a**) the cumulative cases of COVID-19 in Italy as of May 4, 2020, and (**b**) evolution in time of susceptibles, detected and hospitalized cases, and deaths until the release of control measures on May 4, 2020. Simulations show the populations in region A and region B for different scenarios: (baseline, blue curves) the setting as of fit in Fig. 6; For all other scenarios severe restrictions (parameter set as of March 22) are in place from the lockdown time: (SC2) lockdown on March 9 (SC2a, red) with and (SC2b, yellow) without fleeing population at lockdown; (SC3) lockdown anticipated to March 2 (SC3a, magenta) with and (SC3b, green) without fleeing population at lockdown.

To parametrize model (1) taking into account the numerous intervention measures adopted in the early phase of the outbreak we define three time periods: before lockdown (February 24–March 8), under initial lockdown measures (March 9–21) and under stricter lockdown measures introduced later (March 22 – May 4). At the lockdown time *T* (March 9), we assume that 0.5% ($\phi$ = 0.005) of the unconstrained population in region A (roughly 130,000 people) migrate to region B. Given the available data, the parameter $\phi$ cannot otherwise be reliably estimated. Nevertheless, the estimated number is plausible, given the reported large migration from the north of the country^5,6^. The model parameters for the two regions were estimated for each time period independently. When available, the estimated parameters from the previous time period were given as initial guesses for the optimizer in the next time period. The estimated and fixed model parameters for the three time periods are summarized in the Supplementary Material. The result of the model fit is given in Figure 6. The parameter estimation results highlight a clear reduction in the contact rates ($\beta _U$), lowering the basic reproduction number in region A from ${\mathcal {R}}_0=3.7$ at the beginning of the outbreak to ${\mathcal {R}}_t=0.76$ at the end of March, in agreement with estimates obtained by other groups^31^. The decrease in the hospitalization rate $\eta$ and increase in the mortality rates $\delta _I,\, \delta _H$ can be explained with the quickly rising number of hospitalizations and deaths in the second half of March and beginning of April. Fitting data for both region A and region B, we clearly see that control measures were effectively in place also in region B, in which the reproduction number was also lowered to ${\mathcal {R}}_t=0.7$ at the end of March. Compared to the abstract scenarios considered in the previous section, we are here in a setting where the population in region B is about 1.5 times larger than in region A, however not fully disease-free at the time of the outbreak. First cases outside of region A were reported at the end of February, quickly isolated, and could be traced back to clusters in Lombardy or Veneto^3^. While control measures were introduced more severely in region A, awareness and other major interventions such as school closure were applied on the whole Italian territory even before the lockdown on March 9. After May 4, the lockdown was released and most severe control measures were relaxed in more steps. As the focus of this work was concerned with the isolation of a specific region and not with the full parametrization of the ongoing outbreak, we do not consider here the relaxations of the control measures, nor data after May 4. Starting from the obtained data-fit, we simulate different possible scenarios for a hypothetical intervention with stricter and/or earlier measures. We compare (Fig. 7a) the number of cumulative cases in region A and region B as of May 4 for different scenarios, with *baseline* representing the parameter setting as of the fit in Fig. 6. For all other scenarios we assume that most severe restrictions (parameter set as of March 22, cf. Supplementary Material) are in place from the lockdown time. In scenario 2 (SC2) we keep the lockdown time as for the data fit and assume that strict intervention measures are in place from March 9, whereas in scenario 3 (SC3) we advance the lockdown by one week to March 2. In both scenarios we consider a fraction (0.5%) of unconstrained individuals (a) to not flee (b) to flee region A immediately before the lockdown. Figure 7b reports the evolution in time of different compartments in region A and B. As the fleeing rate is assumed homogeneous among compartments (cf.  condition (3)), the migration from region A to region B is most evident in the susceptible population, which at the time of the lockdown is yet very large. The projected scenarios show that stricter interventions from the beginning of the lockdown could have reduced the number of cumulative cases as of May 4 by at least one third, and that importation of cases has far less impact than timely intervention.

### Sensitivity analysis

**Figure 8:**
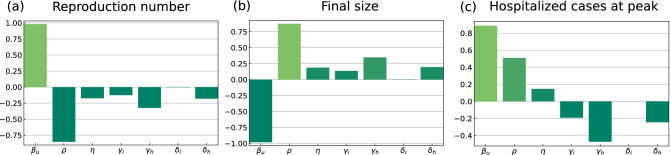
Partial rank correlation coefficients (PRCCs) of seven main model parameters for (**a**) reproduction number, (**b**) final size (${\varvec{S_\infty }}$) and (**c**) the number of hospitalized cases at peak in region A. Parameters with PRCC larger than zero are positively correlated with the quantity of interest, whereas parameters with negative PRCC are negatively correlated. Parameters were varied within the ranges given in the Supplementary Material.

To quantify the effect that model parameters, in particular those which might be affected by control measures, have on critical quantities of the epidemic, we performed a global sensitivity analysis using PRCC on seven model parameters (cf. “Methods” and Supplementary Material). Figure 8 shows the result of the sensitivity analysis of the reproduction number ${\mathcal {R}}_0$, the final size ($S_{\infty }$) as of formula (4), and the number of hospitalized cases at the outbreak peak in the target region A (analogous results for region B, not shown here). Panels in Fig. 8 show that $\beta _U$ and $\rho$ have the strongest correlation with all the three model outputs. Therefore, a small increase in the transmission rate of undetected cases or a small decrease in the detection ratio can significantly increase the spread of the disease (${\mathcal {R}}_0$) and the number of hospitalized cases at the outbreak peak eventually leaving much fewer susceptibles in the population at the end of the epidemic ($S_{\infty }$). Recovery, hospitalization and death rates are negatively correlated with the reproduction number (positively with $S_{\infty }$). The number of hospitalized cases at the outbreak peak is also strongly affected by the recovery rate for the *H* compartment ($\gamma _H$).

## Discussion

A major epidemic outbreak might require authorities to introduce various control measures, from encouraging social distancing to closing educational and economical activities. The goal is to limit the number of victims and to prevent the health care system from collapsing. In the present study, we have established a model for the spread of an epidemic outbreak considering the effects of an exodus of large masses of people from infected areas before restrictive control measures are introduced. Such a phenomenon was reported in the occasion of the COVID-19 outbreak, first in Wuhan, China and then in Northern Italy. The compartmental model introduced in our work differentiates infected people based on the severity of their infection, accounting also for presymptomatic transmission, undetected infections and hospitalized cases. We consider two geographic regions, with the disease being initially confined in region A, where also strict control measures are applied and from where people flee to a previously (ideally) uninfected region B. In contrast to previous studies for the spread of COVID-19, we consider here the geographic distribution of cases, but relax the classification of the individuals into age groups^24,32,33^. Calculating the basic reproduction number (2), we identified its four components that account for transmissions from presymptomatic, undetected infectives, detected but not hospitalized, and hospitalized cases. Further we have derived a new type of final size relation (4), a formula serving for the estimation of the total number of infectives during the whole duration of the epidemic. A final size formula in nonautonomous epidemic models was previously obtained, for example, in a model for Ebola Virus Disease^34^. The novelty in this work is that the final size relation obtained here covers models in which not only the parameters, but also the population size varies at some specific time point *T* (in the considered example due to a fleeing fraction of individuals from one region to another). The result presented here is novel and flexible, so that it is suitable to study various outbreaks of infectious diseases. Further, the same approach could be used for describing migration out of region A and distribution of a fraction $\phi _i$ of individuals moving from region A to a region $B_i$, $i=1,\ldots , n$ for an arbitrary number $n \in {\mathbb {N}}$ of destination regions.

By means of numerical simulations, we have assessed the short-term effect of various parameters on the number of cases and the number of deaths. Figures 2, 3, 4 and 5 show that the earlier the lockdown happens, the slower and less dramatic the epidemic evolves, in agreement with previous studies^7–9^. A late lockdown importantly advances the peak in the number of currently infected people and leads to significantly more deaths and total cases. The fraction of people moving from region A to region B, however, rather affects the latter region. The more individuals moving from region A to region B, the earlier the peak in the infective curve of the target region. The strength and timing of intervention measures in region B is crucial to contain the number of cases and deaths due to importation of the disease from region A (Fig. 3b). We have first assumed that control measures are applied at the same time of the lockdown both in region A and in region B, simulating the behavior of people fleeing isolation and joining their relatives, rather than avoiding restrictive measures. Delays of up to a month in intervention in region B, compared to the lockdown time of region A, can be balanced out with the maintenance of stricter control measures also in region B. More restrictive measures postpone (and lower, cf. Fig. 3b) the peak of the outbreak, however this prolongs the time intervention measure should be in place, if reaching the peak of the infections curve is the criterion for controls to be relaxed.

As an example, we have fitted our model to the Italian COVID-19 data (Fig. 6), the most affected areas in the country defining region A, and the rest of the country corresponding to region B. Projection of different scenarios (Fig. 7) shows that timely and strict intervention could have importantly lowered the number of cumulative cases (in region A by at least 60%) and that migration at the time of lockdown possibly played a minor role for the spread of the disease in Italy. A limitation of our work is that in our setting, migration from region A to region B is assumed to occur as an impulse at time *T*. Given the available data, the fleeing fraction ($\phi$) could not be reliably estimated and it was fixed to obtain a plausible value for the reported large migration from the north of the country. While it was initially observed that a large number of people fled the quarantined regions immediately before these were confined, it is yet debated if the exodus was not rather diluted over the time starting on February 23^35^. A repeated or continuous migration from region A to region B could be included in the model simulations, though this would increase uncertainty in the parametrization. Similarly, the assumption that all unconstrained compartments move in the same proportion from region A to region B could be relaxed, and different fleeing scenarios could be assumed. In all cases, the final size formula (4) would still hold true, as long as the parameters before/after intervention are known and the values for detected cases, hospitalized, recovered and deceased people at time *T* can be estimated from the early outbreak data. In this study, we focused on the timing and strength of intervention, but did not take into account relaxation of control measures. Our setting is realistic for short-term intervention scenarios, such as two to three months, as was the case in many countries during the COVID-19 pandemic. For long-term predictions, assuming maintenance of strict control measures over several months leads to an unrealistically low estimate for the number of total cases and deaths.

## Supplementary information

Supplementary material 1.
