# Supplementary material for: Fleeing lockdown and its impact on the size of epidemic outbreaks in the source and target regions – a COVID-19 lesson
Source: Sci Rep. 2021 Apr 29;11:9233. doi: 10.1038/s41598-021-88204-9 (PMC8085000; doi:10.1038/s41598-021-88204-9)
Supplement: Supplementary file 1 — Supplementary material 1. [file 41598_2021_88204_MOESM1_ESM.pdf]

Supplementary material

Fleeing lockdown and its impact on the size of epidemic outbreaks in the source and target regions – a COVID-19 lesson

MV Barbarossa*, N Bogya, A Dénes, G Röst, H Vinod Varma, Zs Vizi;
(*) barbarossa@fias.uni-frankfurt.de

Computation of the basic reproduction number $\mathcal{R}_0$

For the analytical computation of the basic reproduction number of the system

$$\begin{aligned}
\dot{S}(t) &= -\lambda(t)S(t) && \text{susceptibles} \\
\dot{E}(t) &= \lambda(t)S(t) - \alpha E(t) && \text{exposed/presymptomatic} \\
\dot{U}(t) &= (1-\rho)\alpha E(t) - \gamma_U U(t) && \text{undetected infectives} \\
\dot{I}(t) &= \rho\alpha E(t) - (\gamma_I + \delta_I + \eta)I(t) && \text{detected, non-hospitalized infectives} \\
\dot{H}(t) &= \eta I(t) - (\gamma_H + \delta_H)H(t) && \text{hospitalized infectives} \\
\dot{R}(t) &= \gamma_I I(t) + \gamma_H H(t) && \text{recovered from detected infection} \\
\dot{R}_U(t) &= (1-\sigma)\gamma_U U(t) && \text{recovered from undetected infection} \\
\dot{D}(t) &= \delta_I I(t) + \delta_H H(t) + \sigma\gamma_U U(t) && \text{deceased,}
\end{aligned} \tag{1}$$

where

$$\lambda(t) = \frac{\beta_E E(t) + \beta_I I(t) + \beta_U U(t) + \beta_H H(t)}{N(t)},$$

and $N(t) = N_0 - D(t)$, N_0 being the total initial population at the beginning of the outbreak. We use the next-generation matrix approach and split the system into a vector of compartments which are going through the infection $x = (E, I, H, U)$ and a vector of non-infectious compartments $y = (S, R, R_U, D)$. Then the system can be rewritten as

$$\begin{aligned}
x_i' &= \mathcal{F}_i(x,y) - \mathcal{V}_i(x,y), \qquad i = 1, \dots, 4, \\
y_j' &= g_j(x,y), \qquad j = 1, \dots, 4.
\end{aligned} \tag{2}$$

In the (x,y)-notation the initial disease-free equilibrium (DFE) of the system is $P_0 = (0, y^*)$, where $y^* = (S_0, 0, 0, 0)$. The linearized system at the DFE can be then written by means of the linearization of $\mathcal{F}$ and $\mathcal{V}$, that is the 4×4-matrices

$$F_{ij} = \frac{\partial \mathcal{F}_i}{\partial x_j}(0, y^*), \qquad V_{ij} = \frac{\partial \mathcal{V}_i}{\partial x_j}(0, y^*),$$

obtaining $x' = (F - V)x$. From the theory of positive matrices, one obtains that the basic reproduction number $\mathcal{R}_0$ is the spectral radius of the next-generation matrix, $K := FV^{-1}$.

For our model we have:

$$F = \begin{pmatrix} \beta_E & \beta_I & \beta_H & \beta_U \\ 0 & 0 & 0 & 0 \\ 0 & 0 & 0 & 0 \\ 0 & 0 & 0 & 0 \end{pmatrix} \quad \text{and} \quad V = \begin{pmatrix} \alpha & 0 & 0 & 0 \\ -\rho\alpha & \gamma_I + \delta_I + \eta & 0 & 0 \\ 0 & -\eta & \gamma_H + \delta_H & 0 \\ -(1-\rho)\alpha & 0 & 0 & \gamma_U \end{pmatrix} \tag{3}$$

Hence

$$FV^{-1} = \begin{pmatrix} \mathcal{R}_0 & * & * & * \\ 0 & 0 & 0 & 0 \\ 0 & 0 & 0 & 0 \\ 0 & 0 & 0 & 0 \end{pmatrix},$$

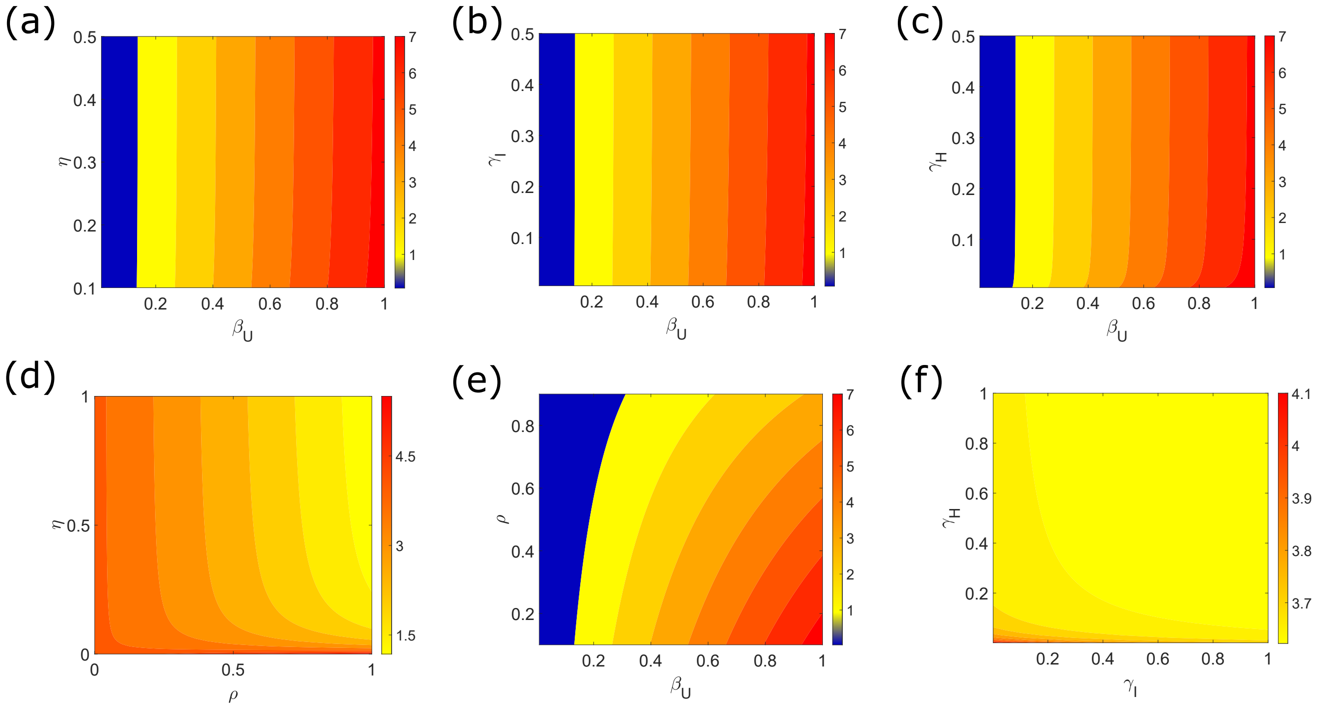

Figure S1. Effect of control measures on the reproduction number $\mathcal{R}_0$. Dependence of $\mathcal{R}_0$ on two combined control parameters, among the transmission rate of undetected infectives (β_U), the recovery rates of hospitalized (γ_H) and non-hospitalized cases (γ_I), the hospitalization rate (η) and the detection probability (ρ). The ratio between β_U and the other transmission rates β_E, β_I and β_H is fixed (cf. Table S2). The reproduction number can be most effectively controlled via regulation of the transmission rate β_U.

where $\mathcal{R}_0$ is

$$\mathcal{R}_0 = \frac{\beta_E}{\alpha} + \frac{\rho\beta_I}{\gamma_I+\delta_I+\eta} + \frac{\rho\beta_H\eta}{(\gamma_I+\delta_I+\eta)(\gamma_H+\delta_H)} + \frac{\beta_U(1-\rho)}{\gamma_U}.$$

Figure S1 visualizes the dependence of the reproduction number on the main control parameters, such as the transmission rate of undetected infectives β_U (assuming one can fix the ratio between β_U and β_E,β_I,β_H respectively), the recovery rates of hospitalized γ_H and non-hospitalized cases γ_I, the hospitalization rate η and the detection probability ρ. While varying γ_I and γ_H, or η and ρ in plausible ranges is not sufficient to drop the reproduction number below the critical threshold $\mathcal{R}_0 = 1$, control can be most effectively achieved via contact reduction, hence regulation of the transmission rate β_U.

Computation of the final size formula

We present in this section all steps necessary to obtain the final size formula for the system (1). Let us assume that for t sufficiently large $N_0 - D(t) \equiv \mathcal{N}$ is constant over time. We define the functions $V(t)$ and $W(t)$ as

$$V(t) = \ln S(t) + a_1U(t) + a_2R(t) + a_3R^U(t) + a_4D(t)$$

and

$$W(t) = \ln S(t) + b_1I(t) + b_2H(t) + b_3D(t) + b_4R(t),$$

where

$$a_1 = \frac{\beta_E}{\mathcal{N}(1-\rho)\alpha}, \quad a_2 = \frac{\beta_I - a_4\delta_I\mathcal{N}}{\gamma_I\mathcal{N}}, \quad a_3 = \frac{\beta_U + \mathcal{N}(+a_1\gamma_U - a_4\sigma\gamma_U)}{(1-\sigma)\gamma_U\mathcal{N}}, \quad a_4 = \frac{\beta_I\gamma_H - \beta_H\gamma_I}{\mathcal{N}(\gamma_H\delta_I - \gamma_I\delta_H)} \tag{4}$$

and

$$
\begin{aligned}
b_1 &= \tfrac{\beta_E}{\mathcal{N}\rho\alpha}, \qquad b_2 = \frac{\beta_U\delta_H + \sigma\gamma_U(-\beta_H + b_4\gamma_H\mathcal{N})}{(\gamma_H+\delta_H)\mathcal{N}\sigma\gamma_U}, \\
b_3 &= \frac{\beta_U}{\mathcal{N}\sigma\gamma_U}, \qquad b_4 = \frac{\left(\frac{\beta_I}{\mathcal{N}} + b_1(\gamma_I+\delta_I+\eta) - b_3\delta_I\right)(\gamma_H+\delta_H) - \eta\left(-\frac{\beta_H}{\mathcal{N}} + b_3\delta_H\right)}{\eta\gamma_H + \gamma_I(\gamma_H+\delta_H)}.
\end{aligned} \tag{5}
$$

The functions V and W are invariants for the system (1). Indeed, differentiation along the solutions yields

$$
\begin{aligned}
\dot V(t) = &-\tfrac{\beta_E E(t)}{\mathcal{N}} - -\tfrac{\beta_I I(t)}{\mathcal{N}} - \tfrac{\beta_U U(t)}{\mathcal{N}} - \tfrac{\beta_H H(t)}{\mathcal{N}} + a_1(1-\rho)\alpha E(t) - a_1\gamma_U U(t) \\
&+ a_2\gamma_I I(t) + a_2\gamma_H H(t) \\
&+ a_3(1-\sigma)\gamma_U U(t) + a_4\delta_I I(t) + a_4\delta_H H(t) + a_4\sigma\gamma_U U(t) \\
= &\, E\left(-\tfrac{\beta_E}{\mathcal{N}} + a_1(1-\rho)\alpha\right) + I\left(-\tfrac{\beta_I}{\mathcal{N}} + a_2\gamma_I + a_4\delta_I\right) \\
&+ U\left(-\tfrac{\beta_U}{\mathcal{N}} - a_1\gamma_U + a_3(1-\sigma)\gamma_U + a_4\sigma\gamma_U\right) + H\left(-\tfrac{\beta_H}{\mathcal{N}} + a_2\gamma_H + a_4\delta_H\right) = 0,
\end{aligned} \tag{6}
$$

and

$$
\begin{aligned}
\dot W(t) = &-\tfrac{\beta_E E(t)}{\mathcal{N}} - \tfrac{\beta_I I(t)}{\mathcal{N}} - \tfrac{\beta_U U(t)}{\mathcal{N}} - \tfrac{\beta_H H(t)}{\mathcal{N}} + b_1\rho\alpha E(t) - b_1(\gamma_I+\delta_I+\eta)I(t) \\
&+ b_2\eta I(t) - b_2(\gamma_H+\delta_H)H(t) + b_3\delta_I I(t) + b_3\delta_H H(t) + b_3\sigma\gamma_U U(t) + b_4\gamma_I I(t) + b_4\gamma_H H(t), \\
= &\, E\left(-\tfrac{\beta_E}{\mathcal{N}} + b_1\rho\alpha\right) + I\left(-\tfrac{\beta_I}{\mathcal{N}} - b_1(\gamma_I+\delta_I+\eta) + b_2\eta + b_4\gamma_I + b_3\delta_I\right) \\
&+ U\left(-\tfrac{\beta_U}{\mathcal{N}} + b_3\sigma\gamma_U\right) + H\left(-\tfrac{\beta_H}{\mathcal{N}} - b_2(\gamma_H+\delta_H) + b_3\delta_H + b_4\gamma_H\right) = 0.
\end{aligned} \tag{7}
$$

In particular, we have the relations $V(T+) = V(\infty)$ and $W(T+) = W(\infty)$, that is

$$
\ln S_{T_+} + \tilde a_1 U_{T_+} + \tilde a_2 R_{T_+} + \tilde a_3 R^U_{T_+} + \tilde a_4 D_{T_+} = \ln S_\infty + \tilde a_2 R_\infty + \tilde a_3 R^U_\infty + \tilde a_4 D_\infty \tag{8}
$$

$$
\ln S_{T_+} + \tilde b_1 I_{T_+} + \tilde b_2 H_{T_+} + \tilde b_3 D_{T_+} + \tilde b_4 R_{T_+} = \ln S_\infty + \tilde b_3 D_\infty + \tilde b_4 R_\infty, \tag{9}
$$

where $\tilde a_j, \tilde b_j$, $j = 1,2,3$ are the coefficients in (5), with values of parameters after intervention at time T. Further we have the relation

$$
R^U_\infty = \underbrace{\frac{(1-\rho)(1-\sigma)}{\rho+\sigma-\sigma\rho}}_{=:\mu}(R_\infty + D_\infty) = \mu\,(R_\infty + D_\infty). \tag{10}
$$

Next we subtract (8) from (9)

$$
\begin{aligned}
&\tilde b_1 I_{T_+} + \tilde b_2 H_{T_+} + (\tilde b_3 - \tilde a_4)D_{T_+} + (\tilde b_4 - \tilde a_2)R_{T_+} - \tilde a_1 U_{T_+} - \tilde a_3 R^U_{T_+} \\
&\quad = [(\tilde b_3 - \tilde a_4)D_\infty + (\tilde b_4 - \tilde a_2)R_\infty - \tilde a_3 R^U_\infty] \\
&\quad \underbrace{=}_{(10)} [(\tilde b_3 - \tilde a_4 - \tilde a_3\mu)D_\infty + (\tilde b_4 - \tilde a_2 - \tilde a_3\mu)R_\infty],
\end{aligned} \tag{11}
$$

and define

$$
A_T := \frac{\tilde b_1 I_{T_+} + \tilde b_2 H_{T_+} + (\tilde b_3 - \tilde a_4)D_{T_+} + (\tilde b_4 - \tilde a_2)R_{T_+} - \tilde a_1 U_{T_+} - \tilde a_3 R^U_{T_+}}{\tilde b_3 - \tilde a_4 - \tilde a_3\mu}, \qquad b := \tfrac{\tilde b_4 - \tilde a_2 - \tilde a_3\mu}{\tilde b_3 - \tilde a_4 - \tilde a_3\mu}. \tag{12}
$$

We observe that

$$
P_T^+ := P(T_+) := \mathcal{N} + D(T_+) \underbrace{=}_{(10)} S_\infty + (1+\mu)R_\infty + (1+\mu)D_\infty, \tag{13}
$$

hence

$$D_\infty = \frac{P_T^+ - S_\infty}{1+\mu} - R_\infty. \tag{14}$$

Solving (11) for D_∞ and using (13) we obtain

$$D_\infty = A_T - bR_\infty. \tag{15}$$

From the relations (14) and (15), and solving for R_∞ we get

$$R_\infty = \frac{P_T^+ - S_\infty}{(1+\mu)(1-b)} - \frac{A_T}{1-b}. \tag{16}$$

We substitute this expression in the relation (15) and obtain

$$D_\infty = -\frac{b(P_T^+ - S_\infty)}{(1+\mu)(1-b)} + \frac{A_T}{1-b}. \tag{17}$$

Now use the relations (17) and (16) in (9):

$$\begin{aligned}\ln S_{T_+} + \tilde{b}_1 I_{T_+} + \tilde{b}_2 H_{T_+} + \tilde{b}_3 D_{T_+} + \tilde{b}_4 R_{T_+} &= \ln S_\infty + \tilde{b}_3\left(-\frac{b(P_T^+ - S_\infty)}{(1+\mu)(1-b)} + \frac{A_T}{1-b}\right) + \tilde{b}_4\left(\frac{P_T^+ - S_\infty}{(1+\mu)(1-b)} - \frac{A_T}{1-b}\right)\\ &= \ln S_\infty + \frac{\tilde{b}_4 - b\tilde{b}_3}{(1+\mu)(1-b)}(P_T^+ - S_\infty) + \frac{(\tilde{b}_3 - \tilde{b}_4)A_T}{1-b}.\end{aligned}$$

Define $\chi = \frac{\tilde{b}_3 - \tilde{b}_4}{(1-b)(\tilde{b}_3 - \tilde{a}_4 - \tilde{a}_3\mu)}$ and $\Phi = \frac{\tilde{b}_4 - b\tilde{b}_3}{(1+\mu)(1-b)}$. Finally we substitute back A_T from (12), use the relation $P_{T_+} = \mathcal{N} + D_{T_+}$. We obtain

$$\begin{aligned}&\ln S_{T_+} + \tilde{b}_1 I_{T_+} + \tilde{b}_2 H_{T_+} + \tilde{b}_3 D_{T_+} + \tilde{b}_4 R_{T_+}\\ &\quad = \ln S_\infty + \Phi(\mathcal{N} + D_{T_+} - S_\infty) + \chi\left(\tilde{b}_1 I_{T_+} + \tilde{b}_2 H_{T_+} + (\tilde{b}_3 - \tilde{a}_4)D_{T_+} + (\tilde{b}_4 - \tilde{a}_2)R_{T_+} - \tilde{a}_1 U_{T_+} - \tilde{a}_3 R^U_{T_+}\right)\\ &\quad = \ln S_\infty + \Phi(\mathcal{N} - S_\infty) + \tilde{b}_1\chi I_{T_+} + \chi\tilde{b}_2 H_{T_+}\\ &\qquad + \left(\chi(\tilde{b}_3 - \tilde{a}_4) + \Phi\right) D_{T_+} + \chi(\tilde{b}_4 - \tilde{a}_2)R_{T_+} - \chi\tilde{a}_1 U_{T_+} - \chi\tilde{a}_3 R^U_{T_+}.\end{aligned} \tag{18}$$

Rearranging the terms we obtain the final size relation as in the main manuscript,

$$\begin{aligned}\ln S_{T_+} - \ln S_\infty = \Phi(\mathcal{N} - S_\infty) + \tilde{b}_1(\chi - 1)I_{T_+} + (\chi - 1)\tilde{b}_2 H_{T_+} + \left(\chi(\tilde{b}_3 - \tilde{a}_4) + \Phi - \tilde{b}_3\right) D_{T_+}\\ + (\chi(\tilde{b}_4 - \tilde{a}_2) - \tilde{b}_4)R_{T_+} - \chi\tilde{a}_1 U_{T_+} - \chi\tilde{a}_3 R^U_{T_+}.\end{aligned}$$

On the example of the model parametrized on the Italian data, and assuming that parameters are maintained in both region A and region B as of the first intervention on March 9, 2020, we verify and visualize in Figure S2 the convergence of the analytical final size formula with the numerical approximation.

Parameter settings in simulations

For numerical investigation of sensitivity of cases and deaths counts with respect to lockdown-time, fleeing fraction and intervention measures (Figures 2–5) the setting in Table S1 was used. Unless otherwise mentioned, initial values for region A are: $E(0) = 100$, $I(0) = 8$, $H(0) = 10$, $U(0) = 100$, $R(0) = R_U(0) = 0$, $D(0) = 2$, and $S(0) = Pop_A - I(0) - D(0) - U(0) - H(0) - E(0) - R(0) - R_U(0)$. In region B the initial population is assumed to be fully susceptible. Further

- In Figure 2(a) $Pop_A = 25e6$, $Pop_B = 50e6$, $\phi = 0.01$ and the lockdown time T varies between one and twelve weeks after the beginning of the outbreak.
- In Figure 2(b) the same setting as for Figure 2(a) is used, but the lockdown time T is fixed at 3 weeks after the beginning of the observations, whereas the migrating fraction ϕ varies between 0.01% and 50%.

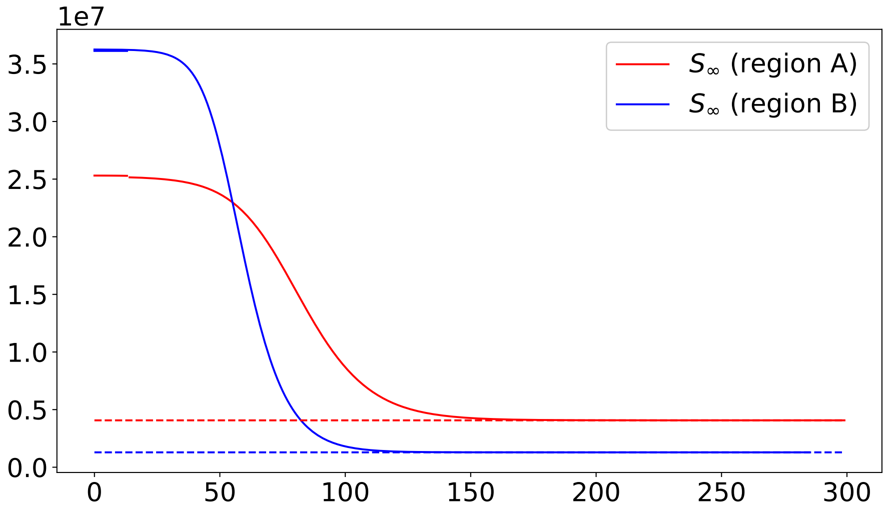

Figure S2. Final size approximation (straight lines) and numerical solution of the susceptible curve of the model (1). Parametrization as for the Italian data (Table S2) assuming parameters remain constant after first intervention measures (March 8) both in region A and region B.

- In Figure 3(a) and Figure 3(b), $Pop_A = 25e6$ and $Pop_B = [0.5, 2, 4] \times Pop_A$. The fleeing fraction of exposed/undetected cases ϕ varies between 0.01% and 50%. Numbers are projected at $t = T + 60$ days and the control measures (reducing the transmission rates by respectively 5%, 30% and 80%) and maintained for two months. Figure 4 is produced with the same setting but running simulations for up to 500 days.
- In Figure 5 $Pop_A = 25e6$ and $Pop_B = [0.5, 2, 4] \times Pop_A$. Region A is isolated on day $T = 21$ since beginning of the outbreak. Intervention in region B occurs T_B days (varying from 3 to 30 days) after T. Cumulative detected cases are projected at $t = T + 90$ days and the control measures in B (reducing the transmission rates by respectively 5%, 30% and 50%) are maintained until the end of the simulations.

For the numerical simulations of the Italian dataset parameter values as in Table S2 were used. Initial values for integration of the ODE system are: for region A, $E^A(0) = 9{,}517$, $I^A(0) = 94$, $H^A(0) = 125$, $U^A(0) = 554$, $R^A(0) = 0$, $R_U^A(0) = 0$ and $D^A(0) = 7$; for region B, $E^B(0) = 486$, $I^B(0) = 0$, $H^B(0) = 2$, $U^B(0) = 5$, $R^B(0) = 1$, $R_U^B(0) = 0$ and $D^B(0) = 0$. Initial susceptible population for both regions were then computed as the difference between the population size and the sum of all other compartments in the region, with $Pop_A(0) = 25{,}307{,}582$ and $Pop_B(0) = 36{,}124{,}240$. For Partial Rank Correlation Coefficients (PRCC) analysis (Figure 8) parameters were varied in the ranges reported in Table S3, and otherwise fixed as in Table S2.

Table S1. Parameter description and values used for numerical simulations of model (1)

**Parameters**	**Value** (unit)	**Description**
β_U	0.5 (1/(days× contact))	Transmission rate from undetected infectives to susceptibles
β_I	0.05(1/(days× contact))	Transmission rate from non-hospitalized cases to susceptibles
β_E	0.01 (1/(days× contact))	Transmission rate from presymptomatics to susceptibles
β_H	0.015 (1/(days× contact))	Transmission rate from hospitalized cases to susceptibles
$1/\alpha$	5.5 (days)[1]	Duration of latency period
γ_U	0.125 (1/days)[3]	Recovery rate for undetected infectives
γ_I	0.1 (1/days)[4]	Recovery rate for non-hospitalized cases
γ_H	0.1 (1/days)[4]	Recovery rate for hospitalized cases
ρ	0.2	Fraction of detected cases (after latent period)
σ	0.001	Probability of detection post mortem
η	0.333 (1/days)	Hospitalization rate
δ_I	0.0004 (1/days)	Disease-induced death rate for non-hospitalized cases
δ_H	0.0167 (1/days)	Disease-induced death rate for hospitalized cases

References

1. an der Heiden, M. & Hamouda, O. Schätzung der aktuellen Entwicklung der SARS-CoV-2-Epidemie in Deutschland – Nowcasting. *Epi. Bull.* **17** (2020).
2. Barbarossa, M. V. *et al.* Modeling the spread of COVID-19 in Germany: Early assessment and possible scenarios. *PLoS ONE* 15(9): e0238559 (2020).
3. Prem, K., Liu, Y., Russell, T. W., Kucharski, A. J., Eggo, R. M. & Davie, N. *The effect of control strategies to reduce social mixing on outcomes of the COVID-19 epidemic in Wuhan, China: a modelling study. Lancet Public Health* **5(5),** e261–e270 (2020).
4. RKI *SARS-CoV-2 Steckbrief zur Coronavirus-Krankheit-2019 (COVID-19)* Robert Koch-Institute, Berlin, Germany 2020. Available online: https://www.rki.de/DE/Content/InfAZ/N/Neuartiges_Coronavirus/Steckbrief.html#doc13776792bodyText11 (last accessed Aug 11, 2020).
5. Istat- Ministero della Salute, *Indagine di sieroprevalenza sul SARS-CoV-2, Anno 2020 (dati provvisori)* available online, Aug 3, 2020. https://www.istat.it/it/files//2020/08/ReportPrimiRisultatiIndagineSiero.pdf

Table S2. Parameter values used for fitting model (1) on Italian data. When multiple rows are given, the first indicates values for region A and the second for region B.

	**24.02 – 08.03**	**09.03 – 21.03**	**22.03 – 04.05**	**Comments**
β_U	0.5091 ± 0.0048<br>0.7016 ± 0.0564	0.2969 ± 0.0056<br>0.4834 ± 0.0208	0.1037 ± 0.0006<br>0.1021 ± 0.0018	estimated
β_I	0.1273<br>0.1754	0.0742<br>0.1209	0.0259<br>0.0255	$= 0.25\beta_U$
β_E	0.1018<br>0.1403	0.0594<br>0.0967	0.0207<br>0.0204	$= 0.20\beta_U$
β_H	0.0509<br>0.0702	0.0297<br>0.0483	0.0104<br>0.0102	$= 0.10\beta_U$
α	1 / 5.5	1 / 5.5	1 / 5.5	assumed[1,2]
γ_U	1 / 7	1/7	1/7	assumed[2,3]
p_H	75%<br>50%	$72.00 \pm 2.57\%$<br>$69.93 \pm 8.81\%$	$49.42 \pm 0.88\%$<br>$33.74 \pm 1.42\%$	estimated
p_{HR}	85%<br>90%	$68.74 \pm 1.46\%$<br>$81.77 \pm 8.60\%$	$78.27 \pm 0.17\%$<br>$83.89 \pm 0.48\%$	estimated
p_{IR}	99%	99%	99%	assumed
γ_I	0.0248<br>0.0495	0.0277<br>0.0298	0.0501<br>0.0656	$= (1 - p_H)p_{IR}/10$
γ_H	0.0850<br>0.0900	0.0687<br>0.0818	0.0783<br>0.0839	$= p_H p_{HR}/10$
ρ	14.5%<br>17.5%	14.5%<br>17.5%	14.5%<br>17.5%	estimated[5]
σ	0.001	0.001	0.001	assumed
η	0.3750<br>0.2500	0.3600<br>0.3496	0.2471<br>0.1687	$= p_H/2$
δ_I	0.0003<br>0.0005	0.0003<br>0.0003	0.0005<br>0.0007	$= (1 - p_H)(1 - p_{IR})/10$
δ_H	0.0150<br>0.0100	0.0313<br>0.0182	0.0217<br>0.0161	$= p_H(1 - p_{HR})/10$

Table S3. Parameter ranges used for sensitivity analysis (PRCC)

**Parameter**	**Range**	**Parameter**	**Range**
β_U	$[0.01, 1]$	ρ	$[0.1, 0.9]$
η	$[0.1, 0.5]$	γ_I	$[0.009, 0.09]$
γ_H	$[0.01, 0.1]$	δ_I	$[0.0001, 0.001]$
δ_H	$[0.005, 0.05]$		
